# Hematopoietic Stem Cell Transplant in Adult Patients with Fanconi Anemia: A Review

**DOI:** 10.3390/diseases13070195

**Published:** 2025-06-25

**Authors:** Bradley Rockwell, Prakriti Ramamurthy, Jhannine Alyssa Verceles, Amanda Lombardo, Amit Verma, Dennis L. Cooper

**Affiliations:** Department of Medical Oncology, Montefiore Medical Center, Albert Einstein College of Medicine, 111 E 210th Street, Bronx, NY 10467, USA; prakriti.sanjay@gmail.com (P.R.); jverceles@montefiore.org (J.A.V.); alombard@montefiore.org (A.L.); amit.verma@einsteinmed.edu (A.V.); decooper@montefiore.org (D.L.C.)

**Keywords:** Fanconi anemia, transplant, conditioning regimens, haploidentical

## Abstract

Fanconi anemia (FA) is characterized by faulty DNA repair and is associated with bone marrow failure, acute myeloid leukemia (AML), and myelodysplastic syndrome (MDS). Because of the more widespread use of next-generation sequencing (NGS) and increased testing for germline mutations in young patients with MDS and AML, FA is increasingly being first diagnosed in adults, many of whom lack classical physical stigmata. Hematopoietic stem cell transplant is the only cure for the hematologic manifestations of FA but there are several unique considerations in FA patients, including first maintaining a high index of suspicion for the diagnosis in patients with minimal phenotypic abnormalities, second an exaggerated sensitivity to alkylating agents and radiation, precluding the use of standard myeloablative conditioning regimens despite the young age of most of the patients, and lastly a marked propensity for squamous cell cancers of the upper aerodigestive tract and anogenital region, likely further increased by the drugs used in conditioning and by chronic inflammation in patients who develop graft-versus-host disease. Despite a growing number of FA patients surviving into adulthood or first being diagnosed with FA as an adult, there is minimal literature describing transplant methodology and outcomes. In the following case-based review of a patient, we incorporate recent findings from the literature on the care of this challenging patient population.

## 1. Introduction/Case Presentation

Fanconi anemia (FA) is a rare genetic, predominantly autosomal recessive disorder characterized by faulty repair of DNA interstrand crosslinks and is associated with bone marrow failure and a marked predisposition to malignancy, especially acute myeloid leukemia (AML) and myelodysplastic syndrome (MDS). Squamous cell carcinomas (SCCs) of the upper aerodigestive tract and anogenital region, including the vulva, are also markedly increased and can even be the first manifestation of the disease [1,2,3,4,5]. The average age at bone marrow failure is 7.6 years and 30–40% of patients will develop a hematologic malignancy by the age of 40 [6]. As children account for most of the reported transplanted FA patients, there has been less consideration of transplant for adult patients with FA, with only one dedicated analysis of adult patients [7] (Table 1). The current relevance of the latter manuscript is limited as the patients were transplanted over a 23-year period from 1991 and 2014 and partially mismatched unrelated transplants comprised only 20% of the cases. Similarly, even recent series generally include only a small number of adolescent and young adult patients, often accumulated over a long period of time, during which time conditioning and graft-versus-host disease (GVHD) regimens evolved, making it difficult to generalize today. Here we review the available literature [7,8,9,10,11] regarding transplant in adult patients with FA by presenting a patient diagnosed with FA who had an atypical genetic work-up and eventually underwent haploidentical (HI) transplant.

*A 29-year-old woman from the Dominican Republic with a history of poorly controlled diabetes and a remarkable family history of an older sister who died from complications related to FA was referred because of cytopenias concerning for MDS. On exam, she had normal stature (170 cm; BSA 2.0) and one café au lait skin lesion. She was pancytopenic with an absolute neutrophil count (ANC) of 1.2 × 10^9^/L, hemoglobin 9.3 g/dL, and platelets 26 × 10^9^/L. A bone marrow biopsy showed a hypercellular (80–90%) marrow with various degrees of dysplasia in the myeloid, erythroid, and megakaryocyte lineages. Cytogenetic analysis showed an abnormal female chromosome complement, with 49 chromosomes showing extra material on 1p, trisomy 1q, gains of chromosomes 3, 9, and 12 and the loss of chromosome 13 in all 14 cells analyzed. The findings were consistent with MDS.*

*Chromosome breakage studies with diepoxybutane (DEB) showed abnormal chromosomal breakage compared to normal but “somewhat fewer than typically associated with FA.” Next-generation sequencing (NGS) showed an autosomal recessive pathogenic mutation in one FANCA gene, with the deletion of Exon 1–3 and a second “likely pathogenic” variant c.3828 in + 2dup exon 38 of the second gene. The abnormal chromosome breakage and NGS studies confirmed the diagnosis of MDS related to FA.*

## 2. Heterogenous Presentation

FA typically presents in the first decade of life with somatic malformations of the skeleton and skin and progressive bone marrow failure, but with a much smaller group of patients presenting for the first time with AML or MDS. The cumulative incidence of MDS or AML is 33% and the risk of any hematologic abnormality is 90% by age 40 [2,6,12].

Probably because of greater awareness and the wider availability of NGS and germline bone marrow failure panels, FA is being diagnosed in adults, who account for about 10% of newly diagnosed patients, many of whom lack the characteristic skeletal and skin abnormalities that often initiate testing in children [13,14]. Apart from a decrease in physical signs, there appear to be other differences in adult FA patients, including a slightly higher proportion of women and an overwhelming predominance of FANCA mutations with a general perception of a milder phenotype, including the retention of fertility in some patients [15]. Adult presentations and atypical DEB-induced chromosomal breakage studies could be due to many factors, including molecular events leading to mosaicism, resulting in partial restoration of the FANC gene function and fewer phenotypic abnormalities [16]. However, it is also possible that the “likely pathogenic” FANCA mutation identified in our patient resulted in a hypomorphic protein with retention of some FANCA function. Ramanagoudr-Bhojappa et al. recently described six FA individuals with delayed development of blood abnormalities (mean age 22 and five 18 years or older) and less than expected chromosome breakage compared to most FA, due to a FANCA variant with reduced but not absent function characteristic of hypomorphic mutations [17]. Similarly, in a study of 227 patients from the Spanish Registry of Patients with FA, patients with less chromosome breakage on DEB had fewer malformations and a later onset of hematologic disease. Notably, about a third of the patients with FANCA mutations in this series had mutations leading to mutant (hypomorphic) protein rather than the absence of protein. From a practical viewpoint, apart from a milder phenotype, the authors noted that patients with hypomorphic mutations may be candidates for strategies that enhance protein function [18].

There are several important considerations in the FA patient, beginning with maintaining a high index of suspicion for the diagnosis, even in patients without the usual physical stigmata. For example, in one study of patients diagnosed or surviving into adulthood, 40% of the patients had no skeletal or skin abnormalities [19]. One of the author’s (DLC) first encounters with FA was a phenotypically normal 24-year-old woman with AML, with complex cytogenetics including abnormalities of chromosomes 1 and 3, who suffered severe toxicity including prolonged myelosuppression after standard idarubicin and cytosine arabinoside induction. Although hemophagocytic lymphohistiocytosis was suspected, a bone marrow failure panel was “mistakenly” ordered and showed FA mutations. Fortunately, the patient eventually recovered following a 7/8 unrelated donor transplant. Abnormalities in chromosomes 1 and 3 should prompt testing for FA in any patient under age 40 (see Table 1) and the marked toxicities after chemotherapy can be unrecognized clues to the underlying diagnosis of FA. In some series the finding of 3q gain or monosomy 7 have been considered to suggest immediate consideration for transplant, while 1 q gain does not appear to be as ominous [20].

Because FA patients show enhanced toxicity to many chemotherapy agents, induction therapy is controversial and patients with AML or MDS are often taken directly to transplant. One study [9] that included 13 adult patients with AML or high-risk MDS described sequential therapy with Fludarabine, cytarabine, and granulocyte colony-stimulating factor (FLAG), followed by reduced intensity transplant without waiting for neutrophil recovery. Progression-free survival and overall survival (OS) were 53% at 3 years but with 2/3 haploidentical transplant patients dying from aspergillus, likely related to the long period of neutropenia (median 42 days from start of FLAG). The 3-year cumulative incidence of relapse was 13%. Interestingly, in this and other studies, active disease seems to result in higher non-relapse-related mortality rather than relapse itself, with the latter averaging about 20–25% in several series [11,21,22]. This suggests that the malignant cells in FA patients are also inherently more sensitive to treatment. As recent retrospective studies have questioned the contribution of downstaging MDS with induction therapy in non-FA patients [23,24], it does not seem that induction therapy is warranted in FA MDS patients, who carry a higher risk for treatment-induced complications. For FA patients with AML, the availability of less toxic induction regimens such as venetoclax plus hypomethylating agents [25] may eventually challenge the paradigm of taking FA patients with AML directly to transplant but there are no mature data in this area at the time of writing.

## 3. Indications for Transplant

Although likely to be a moving target as less toxic conditioning regimens (CRs) are developed [26], currently, the indications for HSCT in FA include severe bone marrow failure (ANC less than 0.5 × 10^9^/L, platelet count less than 30 × 10^9^/L, and a hemoglobin level less than 8 g/dL), progression of moderate bone marrow failure, poor prognostic cytogenetic abnormalities (e.g., 3q+ and 7−), and overt MDS/AML [27]. While chemotherapy versus HSCT has never been compared head-to-head, at this time HSCT is the standard of care for new MDS/AML. Typically, transplant outcomes are more favorable when performed prior to transfusion dependency and initiation of androgen therapy, and before clonal evolution [3]. Gene therapy may eventually become a viable strategy for patients with bone marrow failure but still has remaining logistical challenges, including obtaining enough autologous progenitor cells to be transduced [28,29].

*In our patient, the presence of hypercellular marrow with dysplastic changes and clonal cytogenetic abnormalities, characteristic of MDS, was an indication for transplant.*

## 4. Donor Selection

Donor selection for FA has paralleled that of the general transplant population, starting with the prioritization of matched related donors, with the caveat that in some individuals FA is first discovered when they are evaluated as potential donors for affected siblings [15]. However, since most patients do not have a matched related donor (MRD), and FA patients with a MRD are probably more likely to be transplanted in childhood [30], alternative donors are often required. Matched unrelated donors have traditionally been considered the next best option and a recent large retrospective study of 813 pediatric patients showed equivalent outcomes between MRDs and matched unrelated donors (MUDs) but with poorer outcomes in partially mismatched family and unrelated donors [31]. Importantly for FA adult patients, data are minimal for patients with mismatched donors, especially HI donor transplants. In a large retrospective study of three large FA referral institutions that included 16 adult patients, age ≥ 19 (HR 13.4) and HLA mismatch (HR 4.7) were predictive of adverse outcome in multivariate analysis. By contrast, using T cell depletion (TCD), Satty et al. reported move favorable results that were not significantly inferior in unfavorable risk patients including older age, a diagnosis of AML, or alternative donors [11]. As this study was conducted in a single institution with evolution of the TCD and conditioning regimens over a 22-year period, it may not be easily exportable to other centers and only included one 4/8 matched haploidentical donor. Umbilical cord blood has not been used extensively in adult FA patients [32], mostly because of concerns for delayed engraftment. Cord blood expansion may address this issue but the only available product, omidubicel [33], is approved for patients receiving myeloablative transplant, a non-starter for FA patients. Even in pediatric FA patients, cord blood has fallen out of favor, except from matched family donors [31,34].

*Our patient did not have any ≥7/8 donors in the registry and she had much higher donor specific antibodies (DSAs) against her mother than a healthy sister. Because of the latter and her younger age, her sister was eventually chosen as a HI donor. Prior to transplant, the patient was treated with 4 weeks of rituximab, which was successful in eliminating the DSAs.*

## 5. Conditioning and Graft-Versus-Host Disease

Prior to the recognition of the enhanced toxicity of alkylating agents, the early experience with transplant in FA was characterized by high treatment-related mortality [7]. Conversely, reducing the doses of chemotherapy in CRs placed an increased emphasis on total body irradiation (TBI) to avoid rejection, which was also associated with an excess of short- and long-term complications. The introduction of fludarabine represented a pivotal advance in FA for promoting engraftment and decreasing mortality, while allowing for the development of reduced-radiation-dose and even radiation-free regimens [35]. Fludarabine is now a core element for most FA preparative regimens and has allowed a great reduction in the dose of, if not the need for, radiation, particularly in patients who receive cells from matched donors [31,34]. Cyclophosphamide is also nearly always included in the transplant program but recognition of the excessive toxicity and mortality in FA patients after the high doses used for non-FA patients with severe aplastic anemia [36] has resulted in an evolution of its use and a considerable reduction in the dose [37]. For example, an early study established that a cumulative dose of ≤60 mg/kg of cyclophosphamide was well tolerated in FA but higher doses were associated with excessive acute graft-versus-host disease (GVHD), likely related to increased tissue injury [38]. Subsequently, cyclophosphamide has been used either as part of CRs [39] or more recently as part of GVHD prophylaxis as posttransplant cyclophosphamide (PTCy) [34]. When used in CRs along with fludarabine, busulfan is sometimes added, particularly in patients with clonal disease (with conflicting results) in no or low-dose radiation programs [8,11,39]. However, in the context of PTCy for GVHD prophylaxis for mismatched donors, TBI is included in CRs in order to avoid graft rejection and excessive alkylating agent exposure [40,41]. Although the higher doses of radiation used in earlier FA transplant studies are clearly associated with the development of carcinomas, preliminary evidence suggests that subsequent malignancies were not increased in FA patients at radiation doses ≤ 3 Gy, with the caveat that much longer follow-up is required [42]. Conversely, it is also not proven that CRs incorporating combinations of alkylating agents are less carcinogenic than regimens incorporating lower doses of TBI.

In order to remain below the maximal “safe dose” of cyclophosphamide, FA patients given PTCy have been treated with two doses of 25 mg/kg on days +3 and +4 rather than the original 50 mg/kg × 2 developed by investigators at Johns Hopkins [43,44]. However, a study in India showed high rates of acute GVHD [43] and subsequent studies from Brazil [34] and India [45] showed that the lower dose of PTCy was markedly inferior compared to the same dose of PTCy used in combination with serotherapy (rabbit antithymocyte globulin or alemtuzumab). In the study from Brazil, the addition of serotherapy to PTCy resulted in an improvement in two-year OS from 50% to 82% and decreased acute and chronic GVHD from 78% to 28% and from 67% to 26%, respectively [34]. It is not clear whether the poor results of PTCy alone are due to an enhanced tendency for FA patients to develop GVHD [46] or whether the dose of PTCy is inadequate to prevent GVHD in patients who receive HI peripheral blood stem cells (PBSC) [47]. Notably, the better results with PTCy plus ATG have been achieved in predominantly pediatric patients with essentially no data on this approach with adult patients who require haploidentical donor transplant [34,45].

Abatacept is also used to target donor T cell activation in matched unrelated donors and recently was introduced in transplants specifically for inherited bone marrow failure disorders, including three 7–8/8 children with FA [48]. In the latter situations, it was given for four doses (days −1, +5, +14, and +28). Abatacept has also been added to full-dose PTCy in the context of HI transplant with PBSC in non-FA patients with hematologic malignancies and was associated with <5% grade 3–4 acute GVHD [49]. As abatacept given until day +28 reduces acute but not chronic GVHD [50], Jaiswal et al. showed that extended dosed (days 0, +5, +20, +35, and monthly until 180) abatacept in combination with PTCy and ATG resulted in low rates of acute and chronic GVHD in children and young adults with non-malignant diseases after HI transplant with PBSC [51]. In sum, these studies suggest the addition of serotherapy and abatacept may further augment the efficacy of PTCy without an increase in morbidity, although this is still under investigation.

**Figure 1:**
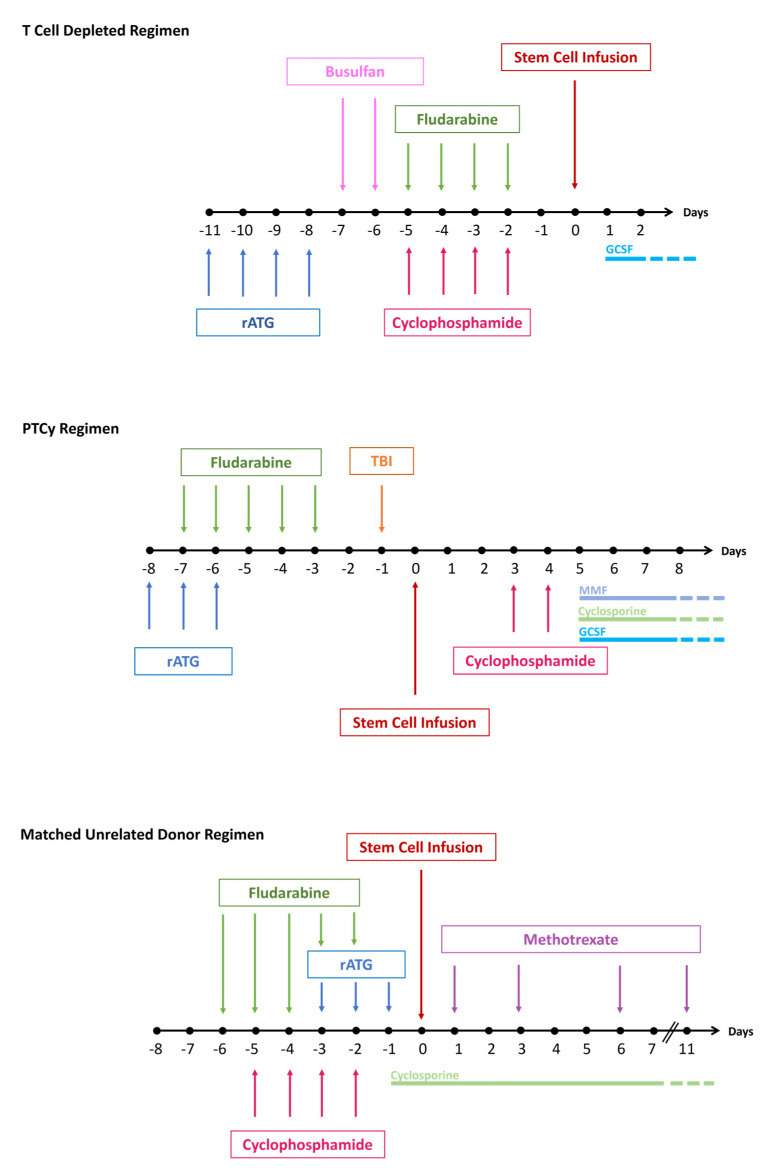
Common conditioning programs for FA. T cell depletion with CD34 selection [11] or TCR αβ depletion is often given with alkylating agent conditioning, often as a radiation-free program without subsequent immunosuppression [11]. The TCD regimens may be used across donor types while the matched donor program is more limited and may not be sufficient for patients with malignant disease (for whom Busulfan or TBI may be added). This regimen has also not been extensively used in adults. When PTCy is given for GVHD prophylaxis, low-dose TBI has been incorporated in conditioning. Although the PTCy platform has been reported exclusively in haploidentical transplants, it may migrate to other alternative donor and even matched donor scenarios as it has in non-FA patients. Notably, all 3 programs include fludarabine and ATG. Timing and individual dosing strategies of ATG continue to evolve. rATG = rabbit antithymocyte globulin, TBI = total body irradiation, MMF = mycophenolate mofetil.

An alternative to PTCy for the prevention of GVHD is the use of ex-vivo T cell depletion (TCD) which has taken the form of CD34+ cell selection and more recently with αβ T cell depletion and CD19 removal to prevent EBV reactivation. When used as GVHD prevention with a fludarabine and TBI CR, TCD showed a 17.4% and 5.5% acute and chronic GVHD, respectively [52]. Notwithstanding the excellent results reported from specialized centers [8,11,52], TCD has not been standardized and recent retrospective studies have suggested that the results with T-replete stem cell products are at least as good as those after TCD, with one study showing a borderline statistically significant negative effect of TCD [10,53,54]. For example, Cancio et al. showed 5-year OS of 73% with TCD and 100% with T-replete transplants [10]. Conversely, a single institution study of predominantly adult FA patients with evolution to MDS/AML showed excellent results with TCD employing CD 34+ cell selection [11]. These results are particularly impressive as more than half received partially mismatched stem cells. Similarly, a recent study from the European Society for Blood and Marrow Transplantation (EBMT) showed that in a pediatric population there were better results with ex vivo αβ TCD than with PTCy after HI transplants [52]. However, in the latter study, it appears that the PTCy patients did not receive serotherapy in addition to lower dose PTCy, which as described above appears to be inadequate. It should also be noted that none of the studies on TCD includes a significant percentage of adult patients with HI donors. Particularly in resource-challenged areas, PTCy plus serotherapy may be the more practical and possibly more effective option than TCD. Three of the major platforms for FA conditioning and GVHD prophylaxis are shown in Figure 1.

**Figure 2:**
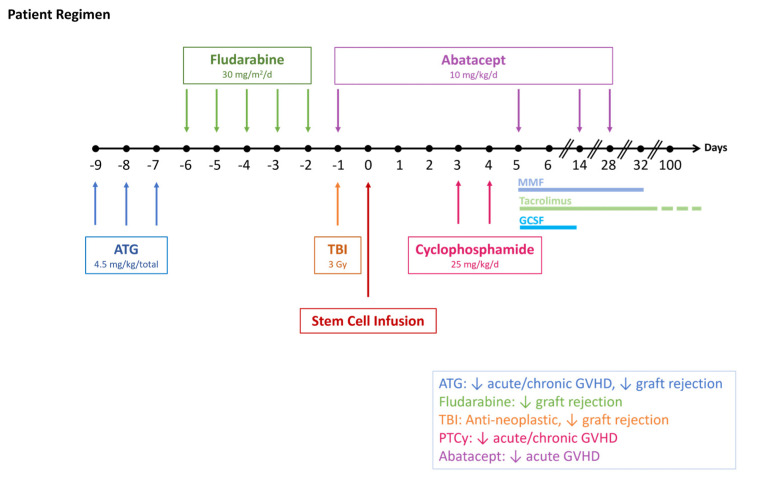
In our patient, abatacept was added to the half-dose PTCy regimen for additional acute GVHD prophylaxis. Recent data suggest that subsequent cancers were less problematic at TBI doses ≤ 3 Gy. As cGVHD may be still problematic, studies incorporating extended dosing abatacept or possibly ruxolitinib may be considered. GVHD = graft-versus-host disease, PTCy = posttransplant cyclophosphamide, ATG = antithymocyte globulin, TBI = total body irradiation, MMF = mycophenolate mofetil.

*For our patient, rabbit ATG 4.5 mg/kg was given over 3 days starting on day −9, Fludarabine 30 mg/m2 × 5 days starting on day −6, and total body irradiation 3 Gy on day −1 for conditioning and PTCy 25 mg/kg on days +3 and +4. She was then started on tacrolimus and mycophenolate mofetil, with the latter stopped at day +30. Abatacept was given on days −1, +5, +14, and +28. (* Figure 2 *).*

*The patient engrafted neutrophils on day +12 and platelets on day +15. Chimerism studies showed 100% donor chimerism for CD3 and CD33 at day 30. The CD4 count was 912 (677–1401) on day +75. She did activate CMV and was treated successfully with valganciclovir. In addition to CMV, she was monitored weekly for Epstein–Barr virus and toxoplasmosis, with no evidence of reactivation.*

*On day +199 she required admission for the explosive onset of multi-organ chronic GVHD, with oral and genital mucosal involvement and liver enzyme elevations. After an unsatisfactory response to steroids alone, she received extracorporeal photopheresis plus ruxolitinib and then belumosudil (because of thrombocytopenia), with a good response.*

As noted above, there are few studies of FA adults who have received haploidentical transplants. Based on the development of severe cGVHD in the current patient, several changes to this program can be considered, including the use of bone marrow rather than PBSC, individualized dosing of ATG [55], more prolonged administration of abatacept as described by Jaiswal et al., or possibly the addition of ruxolitinib for GVHD prophylaxis [51,56]. Based on the results from Satty et al., TCD can also be considered, with the caveat that there are minimal data for adult patients who need an HI donor transplant. For example, in the recent EBMT study [57] describing the superiority of αβ/CD19 depletion to PTCy for HI transplants, 75% of the patients were under the age of 12. In a similar study employing αβ/CD19 depletion, all the patients were <21 years of age and the mega-doses of CD34+ cell dose/kg are not easily achievable for adult patients [52]. Thus, the optimum CR and GVHD prophylaxis for adult patients who require a haploidentical donor remains unknown.

## 6. Long-Term Follow-Up of the FA Patient

Because of improvements in survival after transplant, long-term survival of FA is largely affected by secondary malignancies, which in some but not all series [18,58] increased after transplant. Transplant-associated risk factors include older age (>20) at the time of the HSCT, radiation in the CR, and anything that increases chronic GVHD, including the use of PBSC [59,60]. Despite the prioritization of alkylating agents over radiation therapy during conditioning, it is not clear that they are less carcinogenic. SCCs, primarily of the oral cavity, esophagus, and anogenital area [5,60], are the most common malignancies. The median time to SCC is 7 years after the HSCT, with some studies estimating a 500-fold increased risk in FA patients [61,62,63]. As an increasing number of FA patients are surviving into adulthood and adults now outnumber children on some registries (personal communication, Fanconi Cancer Foundation), the issue of oral and anogenital SCCs is gaining in recognition, a problem that is magnified by the lack of proven chemoprevention and surveillance strategies. Screening guidelines do not specify any changes posttransplant, but regular follow-up with otolaryngology is recommended [27]. As these patients are poor candidates for wide-field radiation and chemotherapy due to excessive toxicity, early detection is crucial, preferably at a premalignant phase, allowing for surgical removal [64].

These data suggest that real progress in reducing late malignancy is likely to require abolishing the use of alkylating agents and radiation in CRs and to eliminate GVHD. Briquilimab, an anti-CD117 antibody that depletes hematopoietic stem cells, was reported recently to be effective as a radiation-free and busulfan-free conditioning treatment in eight patients with bone marrow failure due to FA [65]. Similarly, an anti-CD45 antibody drug conjugate was effective in three different mouse models of FA [66]. With respect to GVHD, optimized TCD or recent graft engineering strategies that provide a product enriched for T regulatory cells may dramatically reduce the duration of, if not the need for, posttransplant immunosuppression [67]. Taken together, the antibody targeting of hematopoietic progenitors and graft engineering strategies offer hope for minimizing conditioning regimen toxicity and GVHD, respectively, in the next decade.

## 7. Non-Malignancy Considerations in the FA Patient

Apart from malignancy surveillance, patients with FA require life-long endocrine care because of an excess of abnormalities of the pituitary stalk, often leading to correctible growth abnormalities with growth hormone, as well as diabetes, osteoporosis, and hypogonadism [68].

*Our patient had a several-year history of diabetes and a fertility consultation before transplant (she had one healthy child) showed very low anti-mullerian hormone, consistent with ovarian failure.*

It is noteworthy that although FA diagnosed in adults appears to have a slightly better survival rate than in children due to a lower rate of bone marrow failure, survivors over the age of 60 remain uncommon [15]. Consistent with health challenges throughout their life, FA patients carry a large psychosocial burden. In a recent study of patients transplanted at a median age of 8.5, only 3 of 58 patients who survived into adulthood were married, few lived independently, held a job, or were married and none had children [69]. A more optimistic outcome was described in a population of FA patients who survived to ≥18 years, 30% of whom had not been transplanted and presumably had phenotypically milder disease. Nearly 90% of the patients had graduated from high school and the majority were working and living independently [19]. These results, while more encouraging, emphasize that comprehensive care of the FA patient does not end with cure of bone marrow failure and surveillance for malignancy.

## 8. Conclusions and Further Studies

HSCT is the only curative option for FA, but data on adult patients are limited. For adult patients who require HI donors, there are essentially no data and results in children cannot be extrapolated to adults. Indeed, even in the pediatric population, patients > 10 years of age have worse outcomes. Data with TCD using αβ/CD19 depletion are intriguing but have been mainly limited to children. Although HSCT outcomes have been improving, the evidence to date suggests that transplant may increase and possibly accelerate the development of SCCs in surviving patients. As the increase in malignancies associated with transplant are potentiated by the agents in the CR and inflammation from GVHD, the next frontier of FA transplant is going to be dominated by the use of monoclonal antibody or antibody–drug conjugate CRs and graft engineering that facilitates engraftment and immune reconstitution while minimizing GVHD. Gene therapy may eventually prove to be effective for patients with bone marrow failure, but as of the time of writing, there are no trials currently recruiting. However, as noted above, these advances will not solve all the medical and social issues that confront the FA patient.

## Figures and Tables

**Table 1:** Transplant outcomes of adult FA patients.

Study	Study Type	Conditioning	Donor Type	GVHD Prophylaxis	Outcome
Bierings et al. 2018 [7]	Retrospective study: 199 adult patients transplanted in EBMT centers 1991–2014	Cy: 96% Flu: 64% TBI: 29%	MSD: 46% MUD: 24% MMUD: 20% UCB: 6%	Details NA but ATG 62% TCD 16%	34% survival but 61% in “ideal” patients transplanted after 2000 with MSD, fludarabine, and bone marrow stem cell source
Mehta et al. 2017 [8]	Prospective study: 45 pts 2009–2014 5 adult pts, 3 with MDS	Flu/Cy/Bu, ATG	MUD: 26 MMUD: 13 MMRD: 6	ATG, TCD (CD34 selection)	80% OS but only 1 of 5 adults survived
Debureaux et al. 2021 [9]	Retrospective study: Sequential chemo and Tx in 18 pts with MDS/AML 2006–2019 median age 22	Flu/Ara-C followed by Cy/Flu/2 Gy TBI	MRD: 2 MUD: 6 MMUD: 1 HI: 3 UCB: 6	CSA + MMF or MTX; PTCy for HI only	53% OS; adult specific survival N/A but 2/3 HI patients died from aspergillus
Cancio et al. 2024 [10]	Retrospective study: 89 pts 2007–2020: 16 adults	Cy/Flu +/− Bu; 12.4% received TBI, 50% adults received TBI	MRD: 12.5% MUD: 50% MMUD: 31.3% HI: 6.2%	ATG, TCD in 41.6% (CD34 selection)	OS: 83.2% 5 yr, 63% > age 10; HR 13.4 for age > 19; 4.7 for mismatch HLA: TCD borderline decrease in OS NRM: HR 29.9 for age > 19
Satty et al. 2024 [11]	Retrospective study: single institution of 30 pts (15 adults ≥ age-20 with AML/MDS)	Cy/Flu + Bu or TBI	MRD: 10% MMRD 30% (4/8–7/8) MUD: 27% MMUD: 33%	ATG + TCD	5 yr OS 67%, 5 yr DFS 54% No difference in OS in patients age > 20; NRM 40% at 5 yrs for patient > 20 but 20% after 2010

^1^ Flu = fludarabine, Cy = cyclophosphamide, Bu = busulfan, TBI = total body irradiation, MSD = matched sibling donor, MUD = matched unrelated donor, MMUD = mismatched unrelated donor, HI = haploidentical, UCB = cord blood, NA = not available, NRM = non-relapse mortality, PTCy = posttransplant cyclophosphamide, AML = acute myelogenous leukemia, MDS = myelodysplastic syndrome, OS = overall survival, HR = hazard ratio, TCD = T cell depletion, ATG = antithymocyte globulin.

## Data Availability

The datasets/patient information generated during and/or analyzed during the current study are not publicly available since they include personal patient information but are available from the corresponding author on reasonable request.

## Abbreviations

The following abbreviations are used in this manuscript:

MDPI	Multidisciplinary Digital Publishing Institute
DOAJ	Directory of open access journals
FA	Fanconi anemia
AML	Acute myeloid leukemia
HSCT	Hematopoietic stem cell transplant
MDS	Myelodysplastic syndrome
NGS	Next-generation sequencing
SCC	Squamous cell carcinoma
GVHD	Graft-versus-host disease
BSA	Body surface area
DEB	Diepoxybutane
CR	Conditioning regimen
ANC	Absolute neutrophil count
MRD	Matched related donor
MSD	Matched sibling donor
MUD	Matched unrelated donor
MMRD	Mismatched related donor
MMUD	Mismatched unrelated donor
HI	Haploidentical donor
UCB	Umbilical cord blood
ATG	Antithymocyte globulin
TBI	Total body radiation
NRM	Non-relapse mortality
PTCy	Posttransplant cyclophosphamide
OS	Overall survival
HR	Hazard ratio
TCD	T cell depletion
DSA	Donor specific antibody
PBSC	Peripheral blood stem cell
EBMT	European Society for Blood and Marrow Transplantation
